# Supplementary material for: Dynamic predictors of COVID-19 vaccination uptake and their interconnections over two years in Hong Kong
Source: Nat Commun. 2024 Jan 4;15:290. doi: 10.1038/s41467-023-44650-9 (PMC10767005; doi:10.1038/s41467-023-44650-9)
Supplement: Supplementary file 1 — Supplementary Information [file 41467_2023_44650_MOESM1_ESM.pdf]

Appendix: Dynamic predictors of COVID-19 vaccination uptake and their interconnections over two years in Hong Kong

Supplementary Table 1. COVID-19 vaccination uptake rates by important determinants in the four periods

Determinants	P1 (Feb 22 – May 28 2021) (% of primary doses vaccine uptake)	P2 (Jun 21 – Dec 16 2021) (% of primary doses vaccine uptake)	P3 (Jan 3 – Mar 10 2022) (% of booster dose vaccine uptake)	P4 (Jun 6 – Nov 17 2022) (% of booster dose vaccine uptake)
Sample size (N)	3523	7056	2580	2020
Gender				
Female	11.8	60.0	20.6	72.8
Male	17.0	65.4	24.0	76.8
Age group (years)				
18-24	6.8	70.6	8.5	66.7
25-34	12.5	61.4	9.6	69.9
35-44	16.5	72.3	23.6	78.4
45-54	21.3	72.6	26.7	80.1
55-64	15.9	64.6	30.8	79.4
65 or above	11.2	49.8	22.0	70.7
Educational attainment				
≤Primary	8.8	45.5	14.1	66.8
Secondary	13.8	62.7	24.2	76.0
≥Tertiary	16.8	69.5	23.1	76.9
Employment status				
Employed/students/home makers/retirees	14.4	62.5	22.1	74.2
Unemployed [a]	8.0	53.6	23.1	76.2
Chronic condition				
With at least one chronic condition	10.2	48.9	20.1	70.4
Without chronic condition	16.1	69.0	23.1	76.9
Married status				
Married	19.5	65.1	25.8	76.9
Divorced/separated/ widowed	12.3	57.7	15.6	70.8
Trust in government [b]				
Strongly disagree	6.9	45.6	16.7	63.3
Disagree	8.9	53.5	21.1	64.9
Even	13.4	62.5	20.9	75.2
Agree	19.4	67.1	26.8	80.7

Strongly agree	26.8	71.3	29.7	81.7

[a] Unemployment group included unemployed persons or who reported that not working for other reasons.

[b] "Trust in government" was identified as an important bridging node in our network analysis. Participants were asked to rate their agreement on the 5-Likert scale on statement of "I believe that the Hong Kong government can take effective measures to control novel coronavirus pneumonia spread in Hong Kong".

Self-reported reasons for non-vaccinations by age groups

In nine survey rounds conducted between 6th December 2021 and 14th July 2022, participants who had not received any / received only one dose / received only two doses of COVID-19 vaccines but indicated that they would be never/very unlikely/unlikely/unsure to take one dose / the second dose / the booster dose of COVID-19 vaccine in the future were asked about the major reasons for being hesitant or resistant about taking COVID-19 vaccination. The open-ended responses for why to be hesitant or resistant about taking a COVID-19 vaccine or a vaccine booster from these nine survey rounds were coded as reasons for vaccination resistance. Participants were asked to provide reasons that first came to mind, then the interviewer jot down notes of participants' statements and asked follow-up questions of "any other reasons" to encourage participants to give more than one reason for their vaccination decision. Amongst the 1043 participants who were eligible to answer the open-ended questions (i.e., they did not take the first dose or the third dose of COVID-19 vaccine), 983 provided at least one reason. Their provided reasons are summarized in the Appendix Table 6.

Supplementary Table 2. Reasons for non-vaccination among participants who did not receive the primary doses and booster dose of COVID-19 vaccine

	Primary vaccine doses uptake (N = 225)						Booster dose vaccine uptake (N = 709)					
Reasons for non-vaccination (%)	18-24	25-34	35-44	45-54	55-64	>65	18-24	25-34	35-44	45-54	55-64	>65
Concerned about vaccine safety	22.20	40.70	56.00	53.30	44.40	33.70	34.20	34.10	26.30	29.60	29.10	31.40
Concerned about vaccine effectiveness	0	14.80	40.00	26.70	11.10	11.90	15.40	23.70	18.60	27.60	22.80	6.90
No urgency or low need to take the vaccine	11.10	22.20	4.00	6.70	5.60	11.90	41.00	30.60	26.30	21.40	27.80	24.50
Concern about chronic condition	11.10	14.70	12.00	40.00	41.70	43.60	1.70	3.50	3.40	5.10	1.30	9.80
Concern about old age	0	0	0	0	2.80	9.90	0	0	0	0	0	5.90
Already got antibodies [a]	33.30	7.40	12.00	6.70	8.30	2.00	12.00	13.30	12.70	8.20	11.40	10.80
Low trust in government	22.20	3.70	8.00	6.70	13.90	0	3.40	1.70	1.70	1.00	7.60	1.00
Lack of social norm or support [b]	11.10	7.40	0	0	11.10	11.90	3.40	1.70	2.50	3.10	1.30	7.80
Perceived low disease risk	11.10	3.70	4.00	6.70	0	5.90	6.80	4.60	5.90	4.10	5.10	5.90
Wait for better vaccine	11.10	0	4.00	0	2.80	1.00	1.70	1.20	3.40	5.10	2.50	2.00
No mandatory / Dislike mandatory	0	0	4.00	0	0	0	7.70	7.50	11.90	5.10	8.90	4.90
Others (i.e., pregnant) [c]	0	3.70	4.00	0	0	0	0.90	2.30	2.50	3.10	2.50	3.90
No incentives to take the vaccine	0	0	0	0	0	1.00	0	0.60	0.80	2.00	2.50	0
Not convenient to take the vaccine	0	0	0	0	0	0	0	0	0	1.00	2.50	0
Free rider psychology [d]	0	3.70	0	0	0	0	0.90	0.60	0	0	1.30	0

[a] Already got antibodies consisted of the following reasons: already got antibodies because of natural infections; antibodies are sufficient after getting two jabs.
[b] Lack of social norm or support consisted of the following reasons: have not been encouraged by doctor/family/friends/other people.

[c] Others consisted of the following reasons: pregnancy; not yet the age to take the third dose; not enough duration after the second jab.
[d] Free rider psychology consisted of the following reason: others will take the vaccine so no need to take it myself.
The blue shaded cells indicate the top three reasons for not taking the primary vaccine doses by the six age groups, while the orange shaded cells indicate the top three reasons for not taking the booster vaccine dose by the six age groups.

Supplementary Table 3. An overview of study survey rounds

Timeframe	Sample size, cooperation rate [a] (N, %)
Period 1 (2021): Covering the fourth pandemic wave and representing an initial period of vaccine roll-out before the introduction of various incentive strategies	
Feb 22 – 23	509 (71.8)
Mar 29 - 31	1001 (64.6)
Apr 27 - 30	1010 (66.6)
May 24 - 28	1003 (62.3)
Period 2 (2021): Covering the post-fourth pandemic wave and representing a scaled-up phase when various incentives were introduced to boost primary doses vaccination uptake	
Jun 21 - 25	1004 (71.2)
Jul 19 – 24	1005 (68.1)
Aug 16 - 20	1006 (68.0)
Sept 13 - 25	1000 (67.2)
Oct 11 - 22	1004 (70.0)
Nov 9 - 18	1011 (72.4)
Dec 6 - 16	1026 (70.0)
Period 3 (2022): Covering the fifth pandemic wave (Omicron wave) and representing a period when the vaccine pass policy was announced and implemented to promote uptake of the booster dose	
Jan 3 - 13	1013 (74.4)
Feb 7 - 10	539 (75.9)
Feb 14 - 17	516 (71.5)
Mar 7 -10	512 (70.3)
Period 4 (2022): Covering the post-fifth pandemic wave and representing a scale-up phase for promoting the completion of the booster dose	
Jun 6 - 15	1003 (59.7)
Nov 7 -17	1017 (61.1)

[a] Survey cooperation rate is defined as the proportions of participants who completed the interviews against subjects who were contacted and identified to be eligible.

Supplementary Table 4. Subthemes and descriptions of corresponding indicators of SVI

SVI subthemes	Descriptions of the indicators [a]
Socioeconomic status [b]	
Poverty	Proportion of persons below poverty line [c]
Unemployment	Proportion of persons aged 15 or above being unemployed
Income	Median income per capita
Educational level	Proportion persons aged 15 or above having education level below high school
Household composition [d]	
Persons aged 65 and older	Proportion of persons aged 65 and older
Persons aged 14 or below	Proportion of individuals aged 14 or below
Single-parent households	Proportion of single-parent households among all households
Elderly living alone	Proportion of elderly (aged 65 or above) living alone
Housing condition [b]	
Household density	Mean number of persons per household
Area of accommodation	Median floor area of accommodation per household
Healthcare system [e]	
Hospital beds	Proportion of hospital beds per 100,000 persons
Intensive care unit (ICU) beds	Proportion of ICU beds per 100,000 persons
Hospital manpower	Proportion of hospital manpower (full-time staff employed by Hong Kong Hospital Authority) per 100,000 persons

[a] All statistics were calculated at the residential district level of Hong Kong.

[b] Data from the 2021 Hong Kong population by-census data. Available from https://www.census2021.gov.hk/en/index.html

[c] Monthly household income that below 50% of the median monthly household income in Hong Kong before any government interventions was categorized as below poverty line.

[d] Data from the 2016 Hong Kong population by-census data due to unavailable access to 2021 by-census reports. Available from https://www.censtatd.gov.hk/hkstat/sub/so459.jsp

[e] Data from the 2020-2021 Hospital Authority Annual Report, Hong Kong. Available from https://www3.ha.org.hk/data/HAStatistics/StatisticalReport/2020-2021

Supplementary Table 5. Details of measures and coding strategy in the MGM networks

Construct level	Node name	Item questions	Network coding
Outcome variable	VA	How many doses of COVID-19 vaccine have you already received?	1 = Taken the first dose<br>2 = Taken the second dose<br>3 = Taken the third dose<br><br>We used the first dose and the third dose vaccine uptake as our main outcomes, non-uptake were coded as 0.
Risk perceptions	RP1	How likely do you think it is that you will contract novel coronavirus pneumonia over the next one month?	1 = Never<br>2= Very unlikely<br>3= Unlikely<br>4 = Evens<br>5 = Likely<br>6 = Very likely<br>7 = Certain
	RP2	How serious or mild do you think novel coronavirus pneumonia would be for you if you were infected?	1 = Very mild<br>2 = Mild<br>3 = Moderate<br>4 = Serious<br>5 = Very serious
	RP3	How much do you worry that you would be infected with novel coronavirus pneumonia in the next one month?	1 = Not at all worried<br>2 = Slightly worried<br>3 = Moderately worried<br>4 = Very much worried<br>5 = Extremely worried
Efficacy	Self_eff	I'm confident that I can take measures to protect myself against novel coronavirus pneumonia.	1 = Strongly disagree<br>2 = Disagree<br>3 = Half-half/ neutral

			4 = Agree<br>5 = Strongly agree
	Tru_gov	I believe that the Hong Kong government can take effective measures to control novel coronavirus pneumonia spread in Hong Kong	Same as above
Health status	PH	Over the past one week, would you say your health is:	1 = Poor<br>2 = Fair<br>3 = Good<br>4 = Very good<br>5 = Excellent
	Stress1	Not being able to stop or control worrying	0 = Not at all<br>1 = Several days<br>2 = More than half the days<br>3 = Nearly every day
	Stress2	Feeling down, depressed, or hopeless	Same as above
	Stress3	Little interest or pleasure in doing things	Same as above
	Stress4	Feeling nervous, anxious, or on edge	Same as above
	Chronic	Has a doctor ever told you that you have any chronic medical conditions?	0 = No<br>1 = Yes
Vaccine confidence attitudes	VC1	(Overall) I think COVID-19 vaccines will be safe	1 = Strongly disagree<br>2 = Disagree<br>3 = Half-half/ neutral<br>4 = Agree<br>5 = Strongly agree
	VC2	(Overall) I think COVID-19 vaccines will be important to have	Same as above
	VC3	(Overall) I think COVID-19 vaccines will be effective	Same as above
	VC4	Receiving COVID-19 vaccines are compatible with my personal value	Same as above
Avoidance behaviours	Avoid1	Avoid going to crowded places	0 = No<br>1 = Yes
	Avoid2	Avoid going out as much as possible	Same as above
	Avoid3	Avoid going to health care facilities	Same as above

	Avoid4	Keep appropriate social distancing with people outside of your family in public places	Same as above
	Avoid5	Avoid using public transportation when not necessary (e.g. going out on holidays)	Same as above
	Avoid6	Avoid social gathering (such as having lunch/dinner)	Same as above
Hygienic behaviour	hygiene	We asked participants how frequent they adopted the below preventive measures:<br>a. Wear face masks when going outside<br>b. Wash hands immediately after going outside<br>c. Avoid touching common objects (e.g. door knobs, lift buttons) or use protective measures when touching these common objects (e.g. cover the object by tissue)<br>d. Wash hands or use hand sanitizer immediately after touching common objects<br>e. Use liquid soap when washing hands<br>f. Rub your hands (surfaces and fingers) with the liquid soap for at least 20 seconds when washing hands	1 = Never<br>2 = Sometimes (less than half of the times)<br>3 = Often (half of the time or more)<br>4 = Always (all of the time)<br><br>Mean score were calculated across all the items. Higher score indicates greater frequency for taking hygienic behaviour.
Cohabit-related variables	Marriage	What is your marital status?	1 = Single<br>2 = Married/ cohabiting<br>3 = Divorced/ separated/ widowed<br><br>For easy interpretation, we combined 1 & 3 and coded as 0 (non-marital status); and coded 2 as 1 (married).
	Live_chi	How many children do you have that is under 12 years old in your family?	0 = None<br>1 = Yes, ____ (provide number)<br><br>For easy interpretation, we treated this variable as binary,

			participants reported to have at least one child were coded as 1.
Demographic characteristics	Sex	Given that we conducted our survey over telephone, interviewee directly recorded participants' gender	1 = Male<br>2 = Female
	Age	Can you provide your age group?	1 = 18-24<br>2 = 25-34 □<br>3 = 35-44 □<br>4 = 45-54 □<br>5 = 55-64<br>6 = 65 or above □
	Education	What is your education level?	1 = Primary or below<br>2 = Secondary school<br>3 = Tertiary or above<br><br>For easy interpretation, we combined 1 & 2 and coded as 0 (Secondary or below), we coded 3 as 1 (Tertiary or above).
	Employ	What is your occupation?	1. Executive and professional<br>2. Clerical and service worker<br>3. Production worker<br>4. Student<br>5. Homemaker / housewife<br>6. Retired person<br>7. Unemployed or not working for other reason<br><br>For easy interpretation, we coded 7 as 0 (unemployed); and combined 1-6 and coded as 1

			(employed or others). This is because we want to strictly distinguish the most vulnerable group from other groups. We defined unemployed as people who self-reported that they have no work or unemployed for a while, rather than people who have no need to seek a job currently (i.e., students, housewife or retired person)[1].
Contextual factors	case_14d	Data obtained from the Hong Kong Centre for Health Protection[2]	In MGM, this variable was treated as a count variable. We averaged the proportion of official-reported confirmed cases prior 14-days of participant's survey date.
	death_14d [a]	Data obtained from the Hong Kong Centre for Health Protection[2]	In MGM, this variable was treated as a count variable. We averaged the proportion of official-reported confirmed cases prior 14-days of participant's survey date.
	SVI5	Where do you live? (provide living district)	We used the percentile ranking method to calculate an overall SVI score for each of the 18 districts in Hong Kong. The SVI score was further categorized into fiver levels:<br>1 = Very low vulnerability<br>2 = Low vulnerability<br>3 = Medium vulnerability<br>4 = High vulnerability<br>5 = Very high vulnerability

			Then we assigned the SVI level (1-5) based on participant's self-reported living district[1,3].

[a] Variable only used in P3 and P4 given the small number of death cases in previous two periods.

Supplementary Table 6. Percentile rank of SVI and its subthemes

District	Socioeconomic status [a]	Household composition [a]	Housing condition [a]	Healthcare system [a]	SVI [b]	SVI level [c]
Central & Western	0.00	0.12	0.12	0.00	0.00	1
Wan Chai	0.06	0.06	0.00	0.65	0.06	1
Eastern	0.24	0.47	0.41	0.65	0.41	3
Southern	0.35	0.35	1.00	0.00	0.35	2
Yau Tsim Mong	0.18	0.18	0.35	0.12	0.12	1
Sham Shui Po	0.76	0.88	0.59	0.47	0.88	5
Kowloon City	0.41	0.41	0.41	0.12	0.24	2
Wong Tai Sin	0.88	0.76	0.76	0.12	0.76	4
Kwun Tong	1.00	1.00	0.88	0.94	1.00	5
Kwai Tsing	0.94	0.82	0.82	0.47	0.94	5
Tsuen Wan	0.24	0.00	0.41	0.47	0.18	1
Tuen Mun	0.71	0.53	0.24	0.82	0.71	4
Yuen Long	0.65	0.88	0.29	0.82	0.82	5
North	0.82	0.71	0.18	0.12	0.47	3
Tai Po	0.47	0.29	0.59	0.12	0.29	2
Sha Tin	0.53	0.59	0.88	0.12	0.65	4
Sai Kung	0.12	0.18	0.59	0.94	0.47	3
Islands	0.59	0.65	0.06	0.65	0.59	3

[a] Percentile rank of the subtheme was generated by the percentile rank of their respective indicators. A higher score (i.e., approaching 1) denoted greater relative vulnerability.

[b] Percentile rank of the SVI was generated by the percentile rank of the sum scores aggregated by the four subthemes. A higher score (i.e., approaching 1) denoted greater relative community vulnerability.

[c] Based on the percentile rank of SVI scores, we further categorized SVI into five levels from 1 to 5: very low vulnerability (0% ~ 20%), low vulnerability (20% ~ 40%), moderate vulnerability (40% ~ 60%), high vulnerability (60% ~ 80%), very high vulnerability (80% ~ 100%).

Measurement accuracy of self-reported vaccine uptake

We compared the actual vaccine uptake rates in Hong Kong reported by the government with the self-reported vaccine uptake rates collected from our repeated telephone surveys to assess the measurement accuracy. Self-reported vaccination uptake rates were generally lower compared to the actual uptake rates during the survey periods (see Appendix Table 4). However, the independent-samples t-test found that there was no significant difference for actual uptake rates (M=0.61, SD=0.33) and self-reported uptake rates (M=0.60, SD=0.31); t(32)=-0.08, p=0.936. Besides, the actual uptake rates were significantly and highly correlated with self-reported uptake rates (r(15)=0.995, p<0.01). Overall, the two assessment indicate that self-reported uptake rates can be used as a proxy for actual vaccination uptake.

Supplementary Table 7. Differences between actual vaccination uptake rates reported by the government and self-reported vaccination uptake rates collected in telephone survey by survey waves

Survey waves	Survey date	Actual vaccination uptake rates reported by the government [a] (%)	Self-reported vaccination uptake rates collected in surveys [b] (%)
1	Feb 22 – 23, 2020	0	0
2	Mar 29 – 31, 2020	461055 (0.08)	106 (0.11)
3	Apr 27 – 30, 2020	916317 (0.15)	170 (0.17)
4	May 24 – 28, 2020	1319759 (0.22)	234 (0.23)
5	Jun 21 – 25, 2021	2092314 (0.34)	381 (0.38)
6	Jul 19 – 24, 2021	3006599 (0.49)	516 (0.52)
7	Aug 16 - 20, 2021	3884763 (0.63)	631 (0.63)
8	Sept 13 - 25, 2021	4451390 (0.73)	701 (0.70)
9	Oct 11 - 22, 2021	4583113 (0.75)	673 (0.67)
10	Nov 9 - 18, 2021	4688635 (0.77)	747 (0.74)
11	Dec 6 - 16, 2021	4823337 (0.79)	744 (0.73)
12	Jan 3 – 13, 2022	5086843 (0.83)	781 (0.77)
13	Feb 7 - 10, 2022	5533139 (0.81)	448 (0.83)
14	Feb 14 - 17, 2022	5782245 (0.83)	445 (0.86)
15	Mar 7 -10, 2022	6376696 (0.92)	466 (0.91)
16	Jun 6 - 15, 2022	6726456 (0.97)	947 (0.94)
17	Nov 7 -17, 2022	6895602 (0.98)	969 (0.95)
Total: M (SD) (%)		61 (33)	60 (31)

[a] Calculated as the actual COVID-19 first dose uptake rates among adults aged 18 or above on the end dates of each survey wave. The total number of eligible populations was adapted based on the government policies that gradually lowered eligible age for COVID-19 vaccine uptake.
[b] Calculated as the proportion of participants who reported they had already received at least one dose of COVID-10 vaccines in each survey wave.

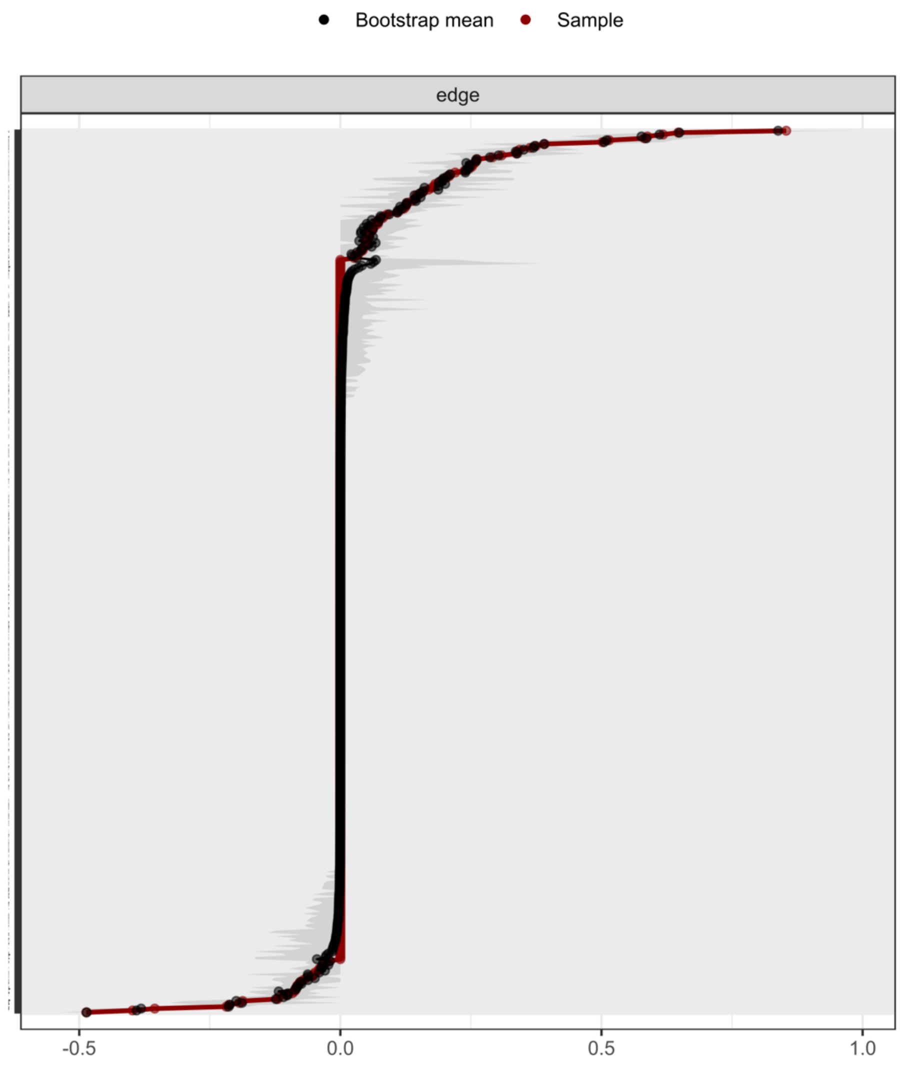

Supplementary Figure 1. Accuracy of the edge-weight parameters and associated bootstrapped confidence intervals (CIs) for the mgm model of P1 network

The red line represents the real sample values; the grey area represents the bootstrapped CIs; all the observed edges of the network were presented on the y-axis but excluded the edge names to avoid information overload. The analysis revealed small and accurate confidence intervals for all edge weights (i.e., the red line falls within the grey area), suggesting that our sample well represents the real population.

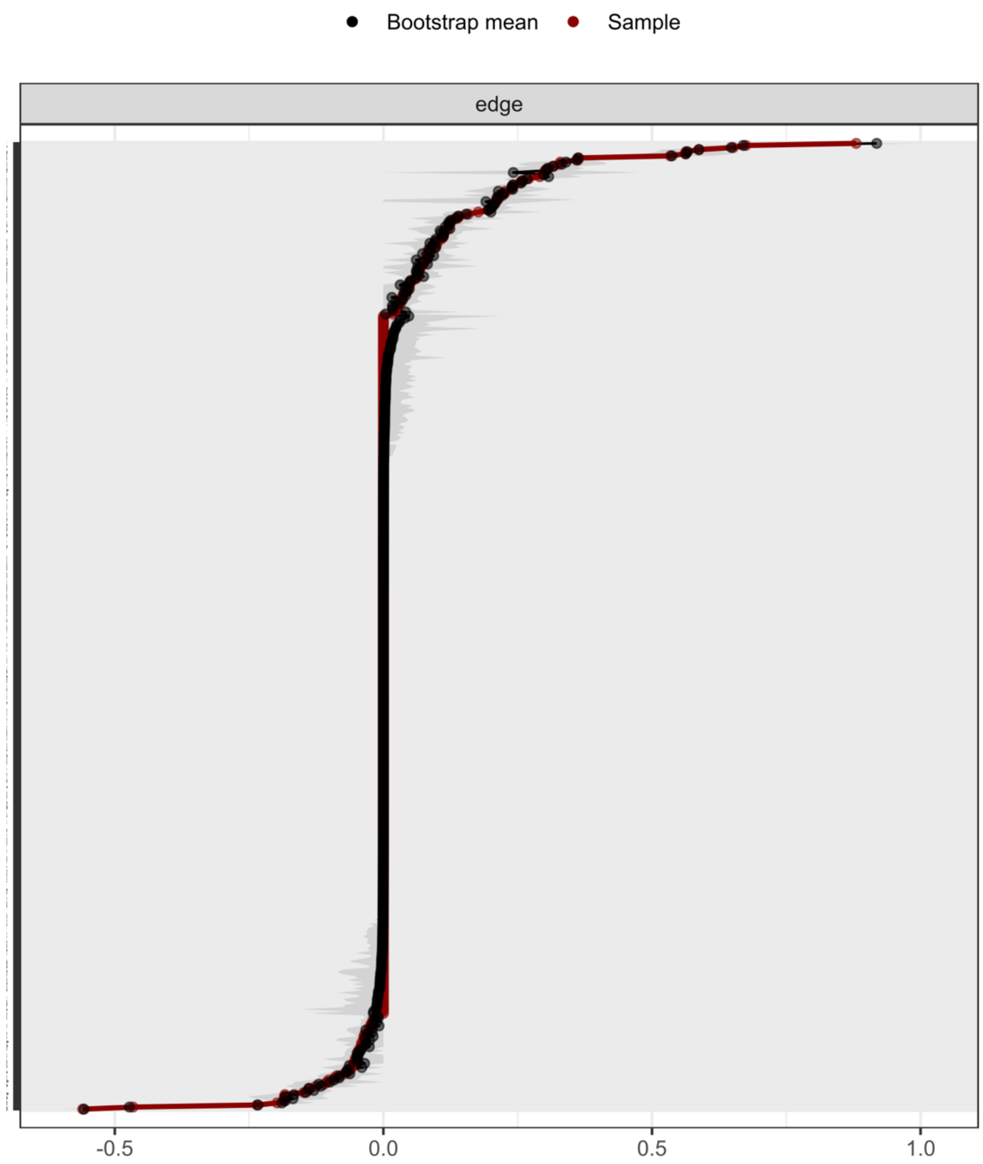

Supplementary Figure 2. Accuracy of the edge-weight parameters and associated bootstrapped confidence intervals (CIs) for the mgm model of P2 network

The red line represents the real sample values; the grey area represents the bootstrapped CIs; all the observed edges of the network were presented on the y-axis but excluded the edge names to avoid information overload. The analysis revealed small and accurate confidence intervals for all edge weights (i.e., the red line falls within the grey area), suggesting that our sample well represents the real population.

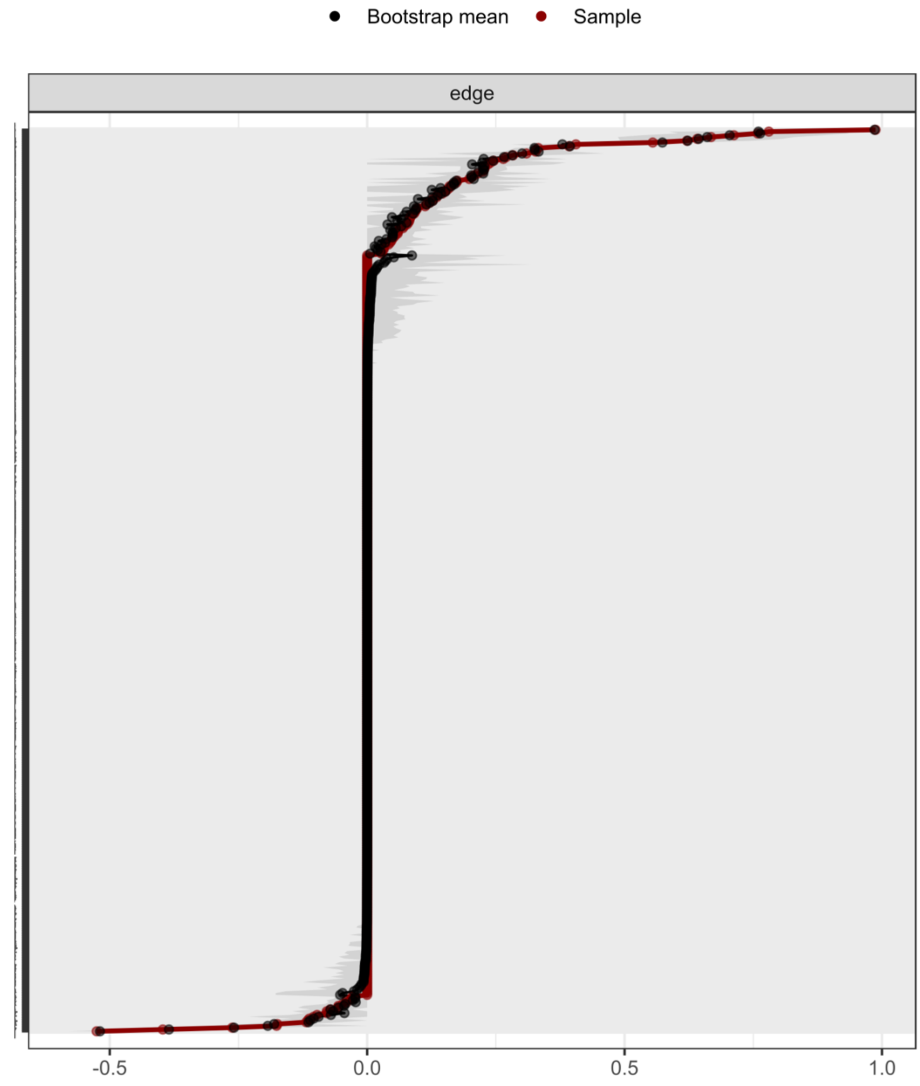

Supplementary Figure 3. Accuracy of the edge-weight parameters and associated bootstrapped confidence intervals (CIs) for the mgm model of P3 network

The red line represents the real sample values; the grey area represents the bootstrapped CIs; all the observed edges of the network were presented on the y-axis but excluded the edge names to avoid information overload. The analysis revealed small and accurate confidence intervals for all edge weights (i.e., the red line falls within the grey area), suggesting that our sample well represents the real population.

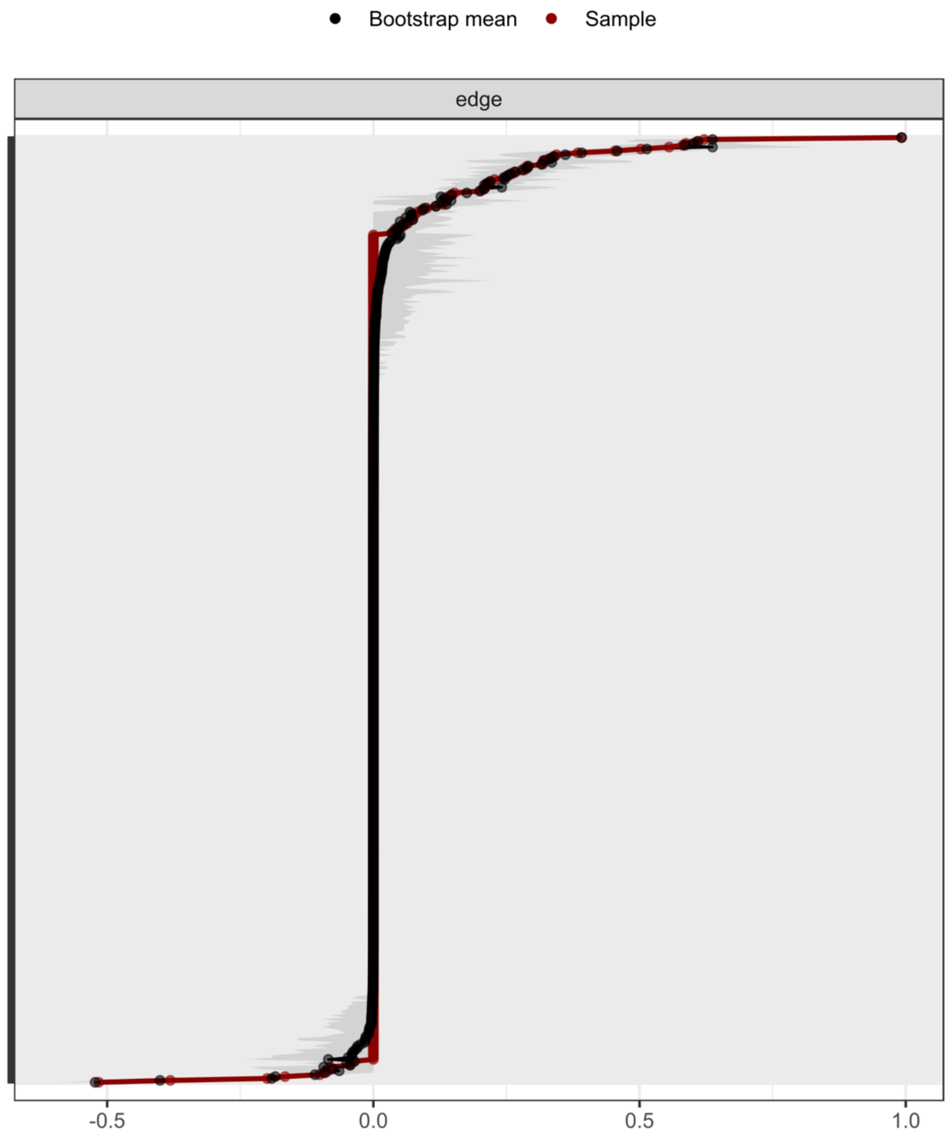

Supplementary Figure 4. Accuracy of the edge-weight parameters and associated bootstrapped confidence intervals (CIs) for the mgm model of P4 network

The red line represents the real sample values; the grey area represents the bootstrapped CIs; all the observed edges of the network were presented on the y-axis but excluded the edge names to avoid information overload. The analysis revealed small and accurate confidence intervals for all edge weights (i.e., the red line falls within the grey area), suggesting that our sample well represents the real population.

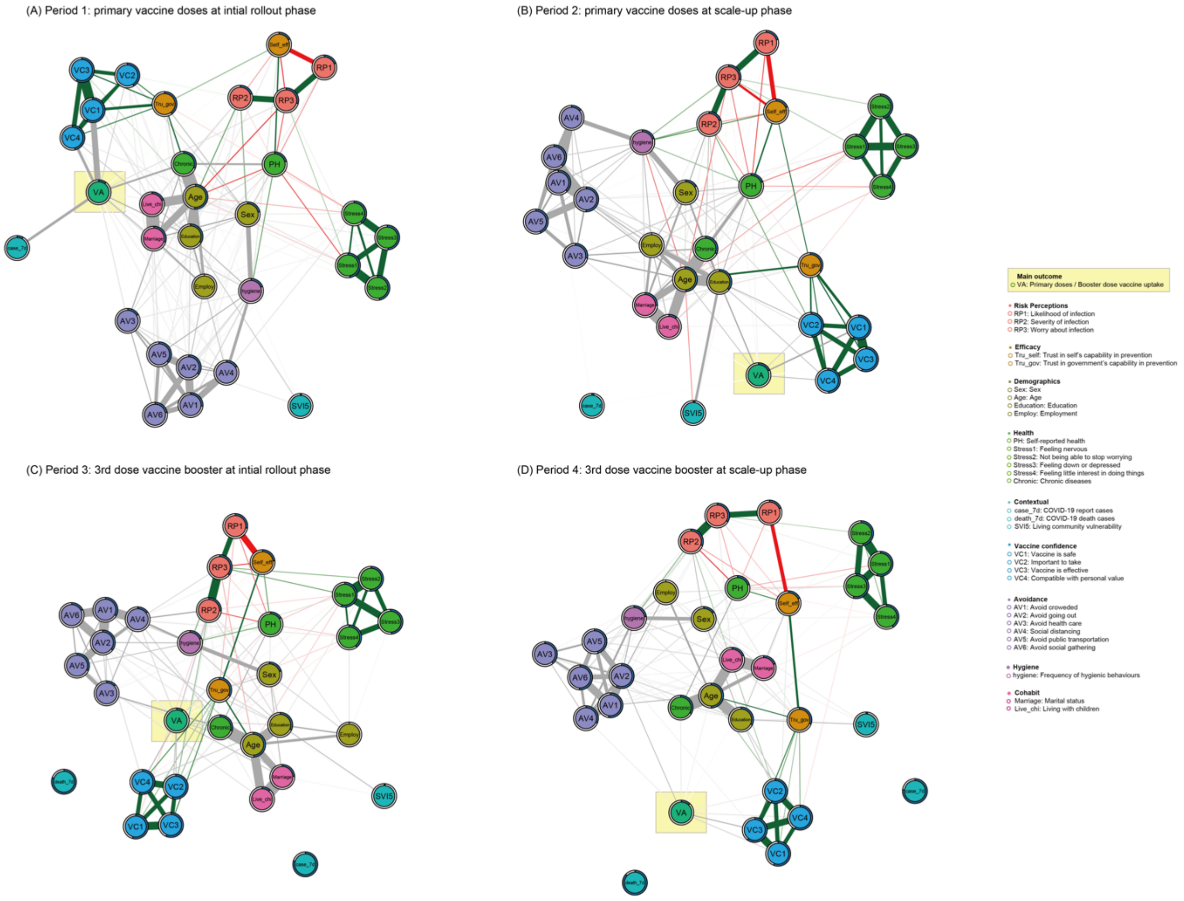

Supplementary Figure 5. Networks of determinants associated with vaccine uptake across P1-P4 using 7-day time window for the COVID-19 report cases and death numbers

This figure replicated our main analyses reported in the main manuscript, except that we replaced the contextual determinants with 7-day time window for the COVID-19 report cases and death numbers. All the variables input in the network across the four periods remained the same, except that number of deaths was included in P3 and P4 but not P1 or P2 due to the extremely low number of human deaths due to COVID-19 in either period. The outcome variable (vaccine uptake) was highlighted in yellow square in each panel figure to ease interpretation. a – d panels show the conditional dependency amongst all the nodes within the network from P1 to P4. We manually categorized all the independent variables into nine domains, as shown with different node colours. Edge thickness indicates the magnitude of the partial correlation between nodes. Green edge represents positive association between continuous variables, red edge represents negative association between continuous variables. No signs were assigned to interaction involving categorical variables, thereby they presented as grey colour. The grey circle surrounding the node indicates the predictability of the node by other nodes.

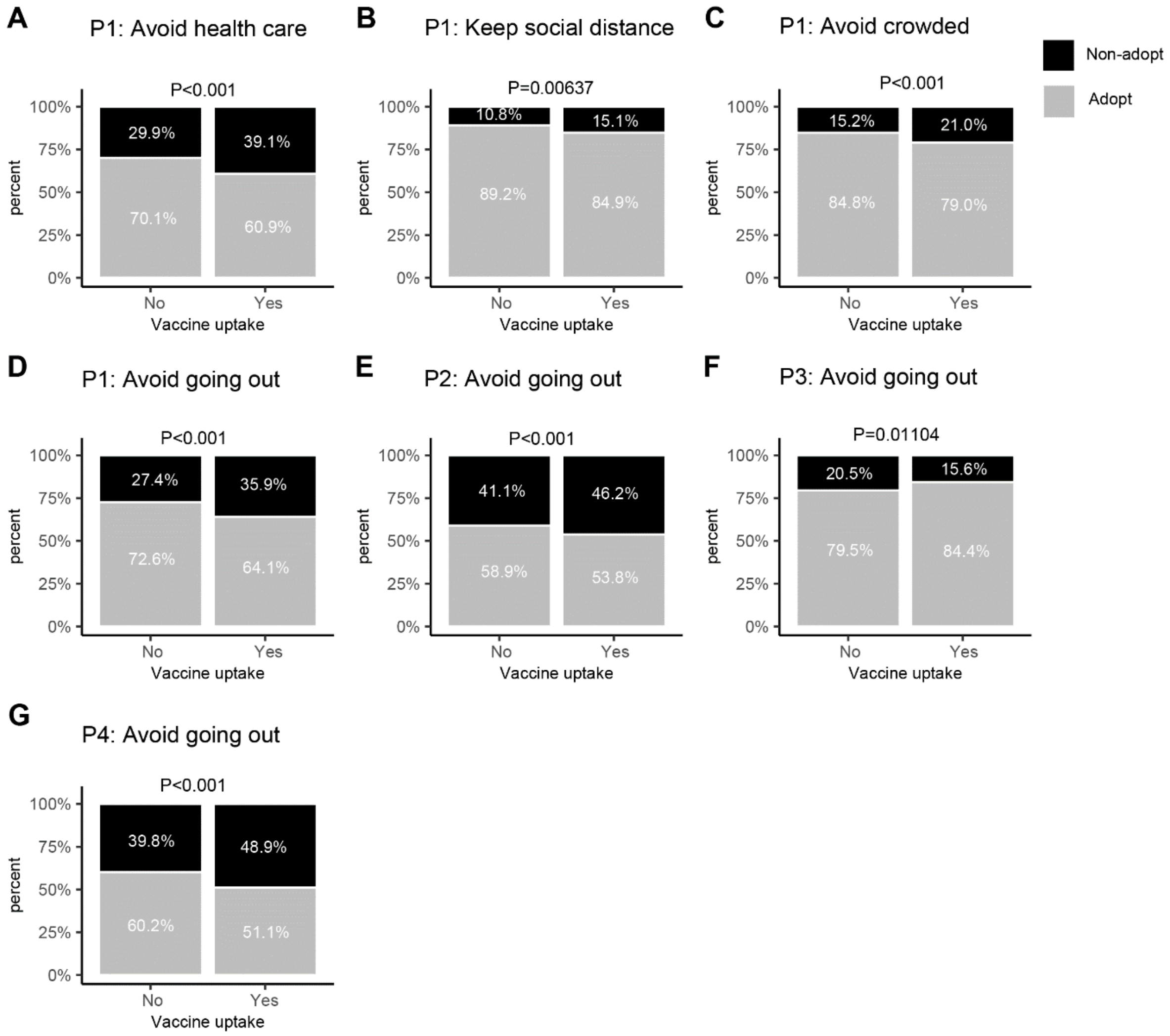

Supplementary Figure 6. Proportion of adopting or non-adopting non-pharmaceutical preventive behaviours by vaccination uptake status

P1 indicates Period 1, P2 indicates Period 2, P3 indicates Period 3, P4 indicates Period 4. P-value was determined by Chi-square test. a-g panels show the major non-pharmaceutical preventive behaviours across P1 to P4. Black block indicates the proportion of participants who reported that they did not adopt the specific non-pharmaceutical preventive behviours, while grey block indicates the proportion of participants who reported that they adopted the behaviours. Major non-pharmaceutical preventive behaviours were selected based on their relative importance to vaccine uptake in mgm networks. From this figure we can rule out the possibility that the negative associations between adoption of non-pharmaceutical preventive behaviours and vaccination uptake could be due to vaccinated people may relax their preventive behaviour. In reality, vaccinated individuals were more inclined to adopt non-pharmaceutical preventive measures. Meanwhile, among the non-vaccinated population, a larger proportion opted for alternative preventive measures for self-protection.

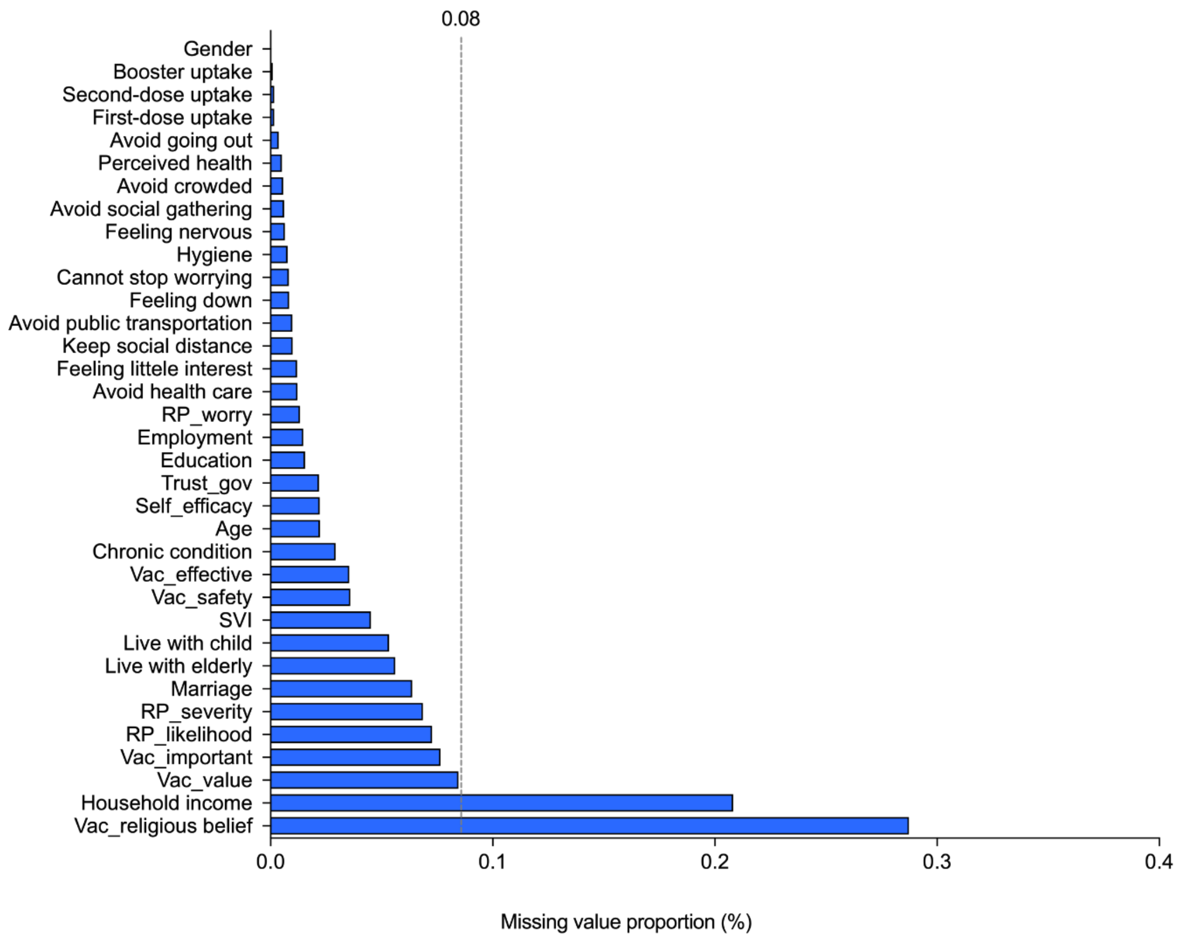

Supplementary Figure 7. Proportion of missing values in selected variables

We removed two variables with high proportion of missing values in the network model, one is monthly household income (missing value rate: 20.8%), another is one of the vaccine confidence items ("I believe that vaccine is compatible with my religious belief"; missing value rate: 28.7%). The high proportion of missing values in these two variables could be that the questions were relatively sensitive to answer over telephone, and not many people can accurately report their household income and religious belief (answer "Don't know" will be categorized as missing in our survey). The remaining variables have no more than 8% (visualized as dotted line) of the missing values were all included into the final models. Abbreviations were used for some variables: "RP_worry" stands for perceived worry of being infected; "Trust_gov" stands for trust in government's capability in pandemic control; "Self_efficacy" stands for trust in personal capability in pandemic control; "Vac_effective" stands for belief in vaccine effectiveness; "Vac_safety" stands for belief in vaccine safety; "SVI" stands for social vulnerability index; "RP_severity" stands for perceived severity of being infected; "RP_likelihood" stands for perceived likelihood of being infected; "Vac_important" stands for belief in vaccine is important; "Vac_value" stands for belief in vaccine is compatible with personal value; "Vac_religious belief" stands for belief in vaccine is compatible with personal religious belief.

References

1 Yuan, J. *et al.* Can psychological distress account for the associations between COVID-19 vaccination acceptance and socio-economic vulnerability? *Appl Psychol Health Well Being* (2023).

2 Department of Health. Data in Coronavirus Disease (COVID-19). https://data.gov.hk/en-data/dataset/hk-dh-chpsebcddr-novel-infectious-agent (2023).

3 Liao, Q. *et al.* Assessing community vulnerability over 3 waves of COVID-19 pandemic, Hong Kong, China. *Emerg Infect Dis.* **27**, 1935 (2021).
